# Retrospective study of 35 cases of breast cancer associated with pregnancy at the Monastir Maternity and Neonatology Center

**DOI:** 10.11604/pamj.2025.52.45.45887

**Published:** 2025-09-29

**Authors:** Asma Korbi, Asma Felfoul, Imen Ben Farhat, Ines Mazhoud, Mounir Ouannassi, Khawla Ben Mohamed, Baraa Aziza, Ahlem Bellalah, Sonia Zaied, Raja Faleh

**Affiliations:** 1 Gynecology Department, Omran Hospital, University of Monastir, Monastir, Tunisia,; 2 Medical Oncology Department, Fattouma Bourguiba Hospital, University of Monastir, Monastir, Tunisia,; 3 Radiology Department, Omran Hospital, University of Monastir, Monastir, Tunisia,; 4 Pathology Department, Fattouma Bourguiba Hospital, University of Monastir, Monastir, Tunisia

**Keywords:** Pregnancy-associated breast cancer, diagnostic delay, clinical characteristics, maternal health, awareness and screening

## Abstract

Pregnancy-associated breast cancer (PABC) is a rare and complex condition, posing significant diagnostic and therapeutic challenges, particularly in resource-limited settings such as Tunisia. This study aims to analyze the clinical and therapeutic characteristics of PABC in a Tunisian population to tailor management strategies. We conducted a single-center, retrospective descriptive study of 35 patients diagnosed between January 2004 and December 2023 at the Monastir Maternity and Neonatology Center. Clinical, radiological, histopathological, and prognostic data were collected and analyzed. The incidence of pregnancy-associated breast cancer was 4.8%. The mean age of patients was 35.3 years, with an average delay of 141 days between symptom onset and consultation. Most cancers were non-metastatic, primarily invasive ductal carcinomas. Management included surgery, chemotherapy, and radiotherapy. Recurrence and mortality rates were 22.9% and 17.1%, respectively. The average overall survival was estimated at 106 months, with better prognosis for younger patients without lymph node involvement. This study emphasizes the importance of increased awareness and improved access to care for these patients, as well as the need to strengthen screening and treatment protocols.

## Introduction

Breast cancer is the most common female cancer in Tunisia and worldwide. Although rare, its association with pregnancy presents major diagnostic and therapeutic challenges [1,2]. The incidence is estimated to be between 1 in 3,000 and 1 in 10,000 pregnancies [3]. Due to physiological changes in the breast, PABC diagnosis is often delayed, complicating early detection [2]. Additionally, this pathology is generally perceived as more aggressive and is detected at an advanced stage [1]. In Tunisia, the management of these patients presents additional challenges due to limited resources [4]. This study aims to examine the clinical and therapeutic characteristics of PABC in a Tunisian population to better tailor diagnostic and treatment strategies for improved patient outcomes and quality of life.

## Methods

**Study design and study setting:** this retrospective, single-center, descriptive study was conducted at the Maternity and Neonatology Center of Monastir, Tunisia. The study period spanned from January 2004 to December 2023.

**Study population:** the study included 35 patients diagnosed with breast cancer either during pregnancy or within one year postpartum. All patients were managed at the study center during the defined period.

**Inclusion criteria:** patients with a confirmed diagnosis of breast cancer during pregnancy or within one year following delivery were eligible for inclusion.

**Exclusion criteria:** patients diagnosed with breast cancer after spontaneous or induced termination of pregnancy were excluded from the study.

**Data collection:** clinical, radiological, surgical, and pathological data were collected retrospectively from patient medical records. Tumor diagnosis was based on clinical examination and/or imaging findings and confirmed by histopathological analysis following either ultrasound-guided core needle biopsy or surgical excision. Tumor characteristics recorded included histological type, lymph node involvement, and immune histochemical markers (estrogen and progesterone receptors, HER2neu status, and Ki-67 index). Tumor staging was carried out according to the 8th edition (2018) of the UICC/AJCC TNM classification.

**Statistical analysis:** all statistical analyses were performed using SPSS software, version 18. Survival outcomes were assessed using the Kaplan-Meier method, and comparisons between groups were made using the Log-Rank test. A p-value of less than 0.05 was considered statistically significant.

## Results

During the study period, 733 breast cancer cases were diagnosed, of which 35 were pregnancy-associated, yielding an incidence of 4.8%. Among these, 54.3% were diagnosed during pregnancy and 45.7% postpartum. The mean age of patients was 35.3 ± 5 years (range: 26-45 years), with 51.4% (n=18) aged 35 years or younger. The majority (65.7%) resided in urban areas. Comorbidities such as hypertension, diabetes, asthma, or hypothyroidism were reported in 2.9% of cases, and 14.3% had a history of prior surgery (Table 1). The mean age at menarche was 12.6 ± 1.2 years, and the mean age at first pregnancy was 27.3 ± 5.2 years. Notably, 31% of patients had their first pregnancy after the age of 30. Breastfeeding was reported by 80% of patients, and 34.3% had used oral contraceptives. A family history of breast cancer was reported in over 20% of cases, and 8.6% had a personal history of breast cancer.

**Table 1:** characteristics of patients with pregnancy-associated breast cancer

Variables	Count (n)		Percentage (%)
**Incidence (pregnancy-associated breast cancer)**	35		4.8%
**Mean age of patients**	35.3±5 years		-
**Urban origin**	23		65.7%
**Medical History**			
**Hypertension**	1		2.9%
**Diabetes**	1		2.9%
**Asthma**	1		2.9%
**Hypothyroidism**	1		2.9%
**Gynecological and obstetric history**			
**Mean age at menarche**	12.6±1.2 years		-
**Mean age at first pregnancy**	27.3±5.2 years		-
**Breastfeeding**	28		80%
**Circumstances of discovery**	**Self-palpation**	24	68.5%
	**Mastodynia**	4	11.4%
	**others**	7	20%
**Average delay between clinical signs and consultation**		141.7±163.1 days	-

The most common presenting symptom was self-palpation of a lump (68.5%), followed by mastodynia (11.4%). The mean delay between symptom onset and medical consultation was 141.7 ± 163.1 days. Among the cases diagnosed during pregnancy, the mean gestational age at diagnosis was 14.9 ± 10.3 weeks; for postpartum cases, diagnosis occurred at an average of 6 ± 3.2 months after delivery. On clinical examination, 84.6% of patients presented with a single breast lump and 15.4% with multiple lumps. Palpable axillary lymph nodes were noted in 36.1% of patients. The right breast was affected in 57.1% of cases, most often in the upper outer quadrant (54.5% on the right and 25% on the left). Imaging via breast ultrasound revealed hypoechoic, irregularly shaped nodules in 76.5% of patients, predominantly located in the upper outer quadrant. The mean size of these nodules was 3.6 ± 1.6 cm (range: 1-8 cm). Mammography showed high breast density in 74.3% of patients, suspicious masses in 80%, and microcalcifications in 42.9% (Table 2).

**Table 2:** management and paraclinical characteristics of breast cancers

Variables	Count (n)	Percentage (%) / Mean±SD
**Breast ultrasound**		
**Single nodule**	22	63.6%
**Hypoechoic nodule**	26	76.5%
**Mean tumor size**	-	3.6±1.6 cm (range: 1-8 cm)
**Tumor location - UOQ**	-	54.5% (right), 25% (left)
**Mammography**		
**Single mass**	21	75%
**High density (ACR C or D)**	26	74.3%
**Microcalcifications**	-	42.9%
**Clinical TNM classification**		
**Stage T2**	13	41.9%
**Stage N0**	23	67.6%
**Distant metastases (liver/lung)**	1	2.9%
**Histology**		
**Invasive ductal carcinoma (IDC, NST)**	-	80%
**Phyllodes sarcoma**	-	n = 1
**Paget’s disease + in situ carcinoma**	-	n = 1
**Immunohistochemistry**		
**HER2 overexpression**	-	25.7%
**Estrogen receptor (ER) positive**	-	50%
**Progesterone receptor (PR) positive**	-	37%
**Treatment**		
**Surgery (mastectomy or lumpectomy)**	31	93.9%
**Chemotherapy**	30	85.7%
**Curative radiotherapy**	29	83.4%
**Timing of diagnosis**		
**Mean gestational age at diagnosis**	-	14.9±10.3 weeks

Staging investigations revealed distant metastases in 2.9% of patients (liver and lungs). The most common histological type was invasive ductal carcinoma (IDC) of no special type (80%). One patient was diagnosed with phyllodes sarcoma of the left breast, and another with Paget’s disease of the nipple associated with contralateral ductal carcinoma in situ. Immunohistochemical analysis showed HER2 overexpression in 25.7% of cases. Estrogen receptor (ER) positivity was noted in 50% of patients, and progesterone receptor (PR) positivity in 37%. Surgical treatment (mastectomy or lumpectomy) was performed in 93.9% of cases, followed by chemotherapy in 85.7% and radiotherapy in 83.4% (Table 2).

**Figure 1:**
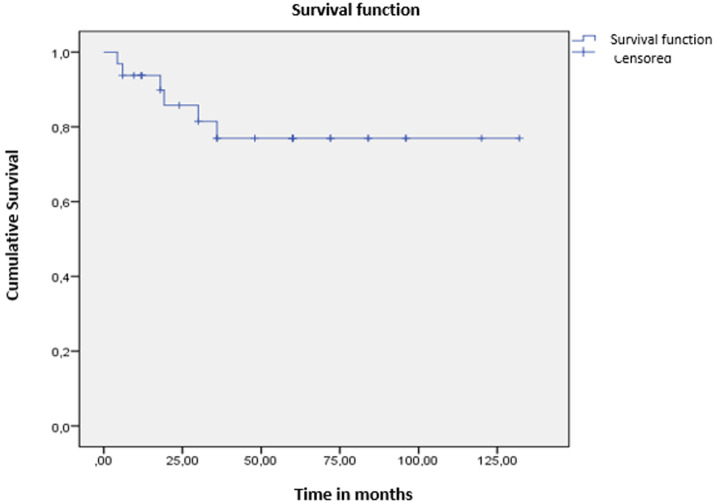
overall survival

**Figure 2:**
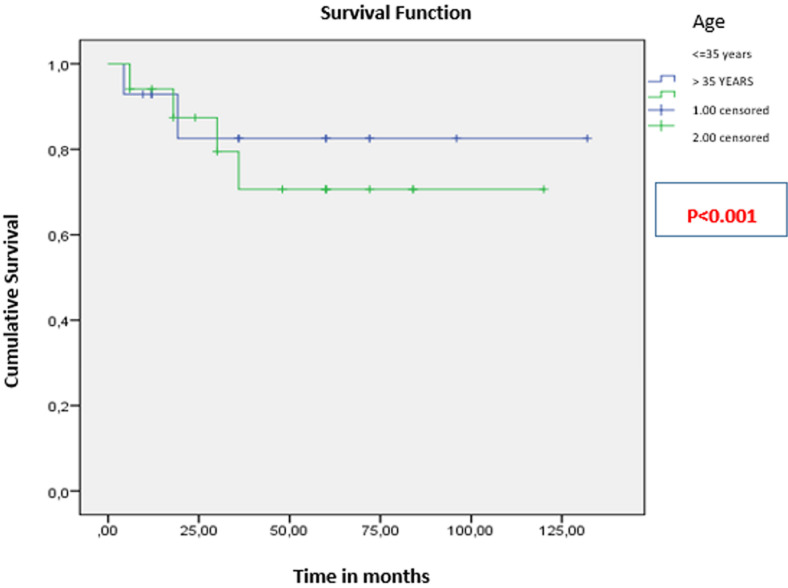
overall survival based on patient age

**Figure 3:**
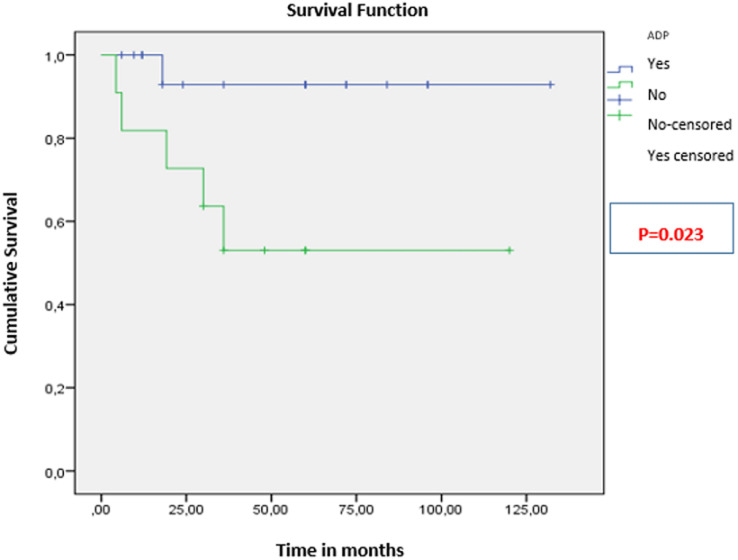
overall survival of patients based on lymph node involvement

**Figure 4:**
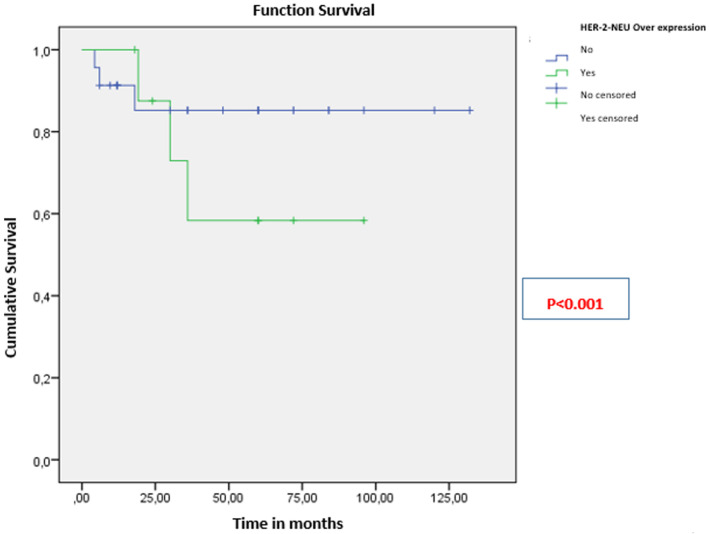
overall survival of patients based on HER2 neu receptor overexpression

The mean overall survival (OS) was 106 ± 9 months (Figure 1). The 3-year OS rate was 81.25%. Patients younger than 35 years had significantly better OS (111 months) than those older than 35 (91 months; p < 0.001) (Figure 2). For patients with clinically detected lymph node involvement at diagnosis, OS was 72 months, compared to 123 months in patients classified as N0 (p = 0.023) (Figure 3). Patients whose tumors overexpressed the HER2 receptor had an OS of 68 months, compared to 114 months in those without HER2 overexpression (p < 0.001) (Figure 4).

Among the 19 patients diagnosed during pregnancy, 15 (78.9%) underwent therapeutic termination. Two patients delivered by cesarean section: one at 34 weeks due to placenta previa and fetal distress, resulting in a live birth; the other at 33 weeks due to fetal distress with absent fetal heart rate, resulting in neonatal death. The remaining two patients delivered vaginally: one at 41 weeks and one preterm at 24 weeks due to premature rupture of membranes following a urinary tract infection.

## Discussion

This retrospective study conducted at the Monastir Maternity and Neonatology Center examined 35 cases of pregnancy-associated breast cancer over 19 years, with a mean age of 35.3 years and a predominance of urban patients (65.7%). Most patients detected the disease through self-palpation of a breast lump. Clinical, ultrasound, and mammography findings revealed hypoechoic, irregular nodules, mostly unifocal and located in the upper outer quadrant. The recurrence rate was 22.9%, and the 3-year survival rate was 81.25%. Our PABC incidence (4.8%) aligns with international data, which reports an incidence around 7% in women under 45 years, with an increase in younger age groups [2]. The mean age of 35.3 years is comparable to other studies in literature, which generally range between 33 and 36 years [5]. The observed early age at menarche (12.65 years) and delay in first pregnancy (27.34 years) are well-known risk factors for breast cancer. Indeed, each year decrease in the age of menarche increases the risk of breast cancer by 5% [6], while a first childbirth after age 30 is associated with an increased risk [7]. Furthermore, multiparity, present in this cohort, is associated with a reduced risk [8].

A palpable mass, found in 57.1% of cases, remains the primary clinical manifestation of pregnancy-associated breast cancer (PABC) [9]. However, physiological breast changes related to pregnancy and breastfeeding can mask early signs of malignancy, contributing to diagnostic delays, which averaged 4.7 months in our study, similar to data from the literature, where delays range from 2 to 15 months [10]. Hard nodules accompanied by palpable lymph nodes in 37.1% of cases align with data showing that PABC often involves significant lymph node involvement. However, our incidence of 36.1% is lower than the rates reported in the literature, reaching up to 92% [11]. Breast ultrasound has been a crucial imaging method, with sensitivity close to 100%, and remains the examination of choice for pregnant women [11]. The BIRADS classification is essential in guiding management, particularly for young or pregnant patients, where mammography is less effective. Contrary to our study results, the literature indicates that PABC is often diagnosed at an advanced stage, primarily due to diagnostic delays [12]. Metastases develop mainly in the lungs, liver, bones, and organs that offer a favorable environment for tumor proliferation due to factors like blood flow and tissue-specific growth signals. Early detection of metastases at these sites is crucial for effective management and to improve patient prognosis [13].

To establish an accurate diagnosis, ultrasound-guided biopsy is recommended in cases of suspected breast cancer, along with lymph node biopsy if suspicious lymph nodes are present. Histopathological examination confirms the diagnosis and evaluates prognostic factors such as the expression of hormone receptors (estrogen and progesterone), HER2 receptor, and Ki-67. It is essential to inform the pathologist that the patient is pregnant [14]. In our study, the predominant histological type was invasive ductal carcinoma. One case of secretory breast carcinoma was observed, a rare form of breast cancer representing 0.03% of breast carcinomas and less than 0.1% of juvenile carcinomas [4,15]. In our study, surgical treatment was performed in 88.6% of patients, including lumpectomies (43.8%) and Patey procedures (56.2%). Lymph node dissection was performed in 82.9% of patients. Curative radiotherapy was administered to 83.4% of patients, and 85.7% received chemotherapy, with 48.6% receiving neoadjuvant chemotherapy. In the literature, surgery is recommended at any stage of pregnancy, as most anesthetic agents are safe for the fetus [16].

Although radiotherapy is theoretically feasible during the first and second trimesters of pregnancy, it is generally avoided due to fetal risks. Chemotherapy based on anthracyclines and taxanes is the standard for PABC treatment during the second and third trimesters. In our study, 22.9% of patients had locoregional recurrences, 11.4% developed contralateral tumors, and 22.9% had metastases. The prognosis of PABC remains controversial; while some authors find no prognostic differences compared to cancers in non-pregnant women when prognostic factors are equivalent, other studies show an increased risk of specific mortality or recurrence [17].

## Conclusion

This study highlights the need for increased awareness and better access to care for this vulnerable population, as well as strengthening screening and treatment protocols specific to pregnancy-associated breast cancer. Future research should explore the long-term impact of treatments administered during this sensitive period and identify new prognostic factors, while also training more healthcare professionals for optimal patient care.

### What is known about this topic

- Pregnancy-associated breast cancer is a rare but clinically significant condition defined by the occurrence of breast cancer during pregnancy or within a year following childbirth;
- It presents complex challenges due to the diagnostic and therapeutic constraints related to pregnancy, as well as concerns for maternal and fetal health.

### What this study adds

- Our study highlights an average delay of 141 days between the appearance of clinical signs and consultation, underscoring a need for awareness to facilitate early diagnosis of breast cancer during pregnancy; these delays can compromise prognosis, and our work emphasizes the importance of increased education for patients and healthcare professionals;
- With a relatively young patient population and few comorbidities, our study describes a specific epidemiological profile characterized by a predominance of invasive ductal carcinoma and non-metastatic tumors;
- By analyzing the outcomes of administered treatments, surgery, chemotherapy, and radiotherapy with recurrence and mortality rates of 22.9% and 17.1%, respectively, our study underscores the importance of a multidisciplinary approach to optimize prognosis.
